# In Vitro Antiproliferative Effects of Benzothiazole-Based Aminosquaraine Dyes Against Cancer Cell Lines

**DOI:** 10.3390/molecules31091537

**Published:** 2026-05-06

**Authors:** Elisabete Alves, João L. Serrano, Ahmed Al-Najada, Sara Cegonho, Vânia Graça, Eurico Lima, Alexandra Varges, Adriana O. Santos, Paulo Almeida, Paulo F. Santos, Samuel M. Silvestre

**Affiliations:** 1 RISE-Health, Faculty of Health Sciences, University of Beira Interior, Avenida Infante D. Henrique, 6201-506 Covilhã, Portugal; 2 RISE-Health, Department of Chemistry, Faculty of Sciences, University of Beira Interior, Rua Marquês de Ávila e Bolama, 6201-001 Covilhã, Portugal; 3 King Abdulaziz City for Science and Technology, P.O. Box 6086, Riyadh 11442, Saudi Arabia; 4 CQ-VR—Chemistry Centre of Vila Real, University of Trás-os-Montes and Alto Douro, Quinta de Prados, 5001-801 Vila Real, Portugal

**Keywords:** aminosquaraine dyes, cytotoxicity, selectivity, apoptosis, cell cycle

## Abstract

The introduction of amine groups into the four-membered central ring of squaraine dyes is expected to enhance the intrinsic cytotoxicity of this scaffold. In this study, the biological effects of eight benzothiazole-based aminosquaraine dyes were investigated by varying the length of the *N*-alkyl chains and the nature of the introduced amine. Biological activity was evaluated through different assays in several cell lines, including apoptosis and cell cycle analysis, as well as confocal microscopy studies. Overall, the incorporation of amino, methylamino, and ethanolamino groups significantly increased cytotoxicity, with the corresponding dyes displaying low half-maximal inhibitory concentration values, in some cases even lower than that of the positive control, 5-fluorouracil. In contrast, derivatives bearing diethanolamino or ethylenediamino groups exhibited lower cytotoxicity, particularly the dye containing *N*-decyl chain. For the dyes selected for further investigation, bearing a methylamino group and *N*-butyl or -hexyl chains, the induction of apoptosis was evident, especially for methylaminosquaraine dye **3**. These dyes also exhibited antiproliferative effects, as evidenced by their ability to induce cell cycle arrest. Confocal microscopy revealed a predominantly cytoplasmic distribution, likely associated with cytoplasmic organelles, with limited penetration into the nucleus. Overall, although squaraines are widely recognized as promising photodynamic agents, the present results suggest that they may also be explored in chemotherapeutic approaches, particularly when exhibiting high intrinsic cytotoxicity.

## 1. Introduction

Following cardiovascular diseases, cancer remains the second leading cause of morbidity and mortality worldwide, exerting a profound socio-economic burden [1]. Despite remarkable scientific advances, the incidence of this multifactorial and highly heterogeneous disease continues to increase globally.

At present, a wide range of drugs is employed in the treatment of malignant tumors, encompassing deoxyribonucleic acid (DNA) intercalating agents (e.g., doxorubicin and epirubicin) [2], topoisomerase inhibitors (e.g., irinotecan, targeting topoisomerase I, and etoposide, targeting topoisomerase II) [3], DNA cross-linking agents (e.g., cisplatin and carboplatin) [4], mitotic inhibitors (including vinca alkaloids, which inhibit microtubule polymerization, and taxanes, which promote microtubule stabilization) [5], antimetabolites [e.g., methotrexate and 5-fluorouracil (5-FU)] [6], and tyrosine kinase inhibitors (e.g., imatinib and erlotinib) [7], among others. However, limited efficacy, severe adverse effects, and the emergence of drug resistance continue to drive the search for more effective therapeutics.

Cyanine dyes constitute a particularly relevant family of molecules. Structurally, they consist of two aromatic nitrogen-containing heterocycles linked by a mono- or polymethine bridge, enabling extensive π-electron delocalization [8,9]. Despite being one of the oldest synthetic dye classes, cyanines remain highly relevant due to their broad applications in biological imaging and optical materials [10,11,12].

Within this broad family, squaraine-based cyanines are 1,3-disubstituted derivatives obtained by condensation of 3,4-dihydroxycyclobut-3-ene-1,2-dione with electron-rich aromatic or heterocyclic methylene bases [13]. Among cyanines, squaraines are particularly distinctive, as the central four-membered ring confers enhanced rigidity and provides a versatile core for functionalization, enabling improved biological relevance [14,15]. Their biological properties can be further tuned by varying the heterocyclic core (e.g., indole- or benzothiazole-based systems) and by modulating hydro-/lipophilicity through adjustment of the *N*-alkyl chain length [16].

In light of the significant antiproliferative activity reported for other non-squaraine cyanines [17], the antitumor potential of a series of squaraine derivatives was evaluated. In the present study, the antiproliferative effects of eight benzothiazole-based aminosquaraines were evaluated in MCF-7 and T47D (human hormone-dependent breast cancer cell lines) and LNCaP (androgen-sensitive prostate cancer cells). To assess tumor selectivity, the compounds were also tested in normal human dermal fibroblasts (NHDF). Optical and fluorescence microscopy, together with flow cytometry, were employed to further characterize their biological effects and to investigate the potential mechanisms of action underlying their antiproliferative activity.

## 2. Results and Discussion

### 2.1. Synthesis

**Scheme 1:**
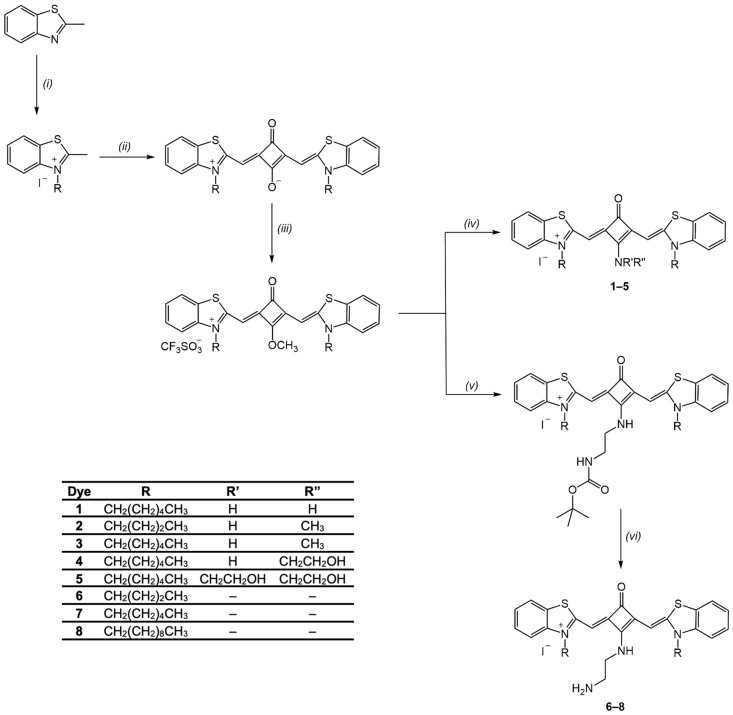
Synthetic route for benzothiazole-based aminosquaraine dyes **1**–**8** and their functional derivatives. Conditions: (*i*) *N*-alkylation of benzothiazole with alkyl iodides under reflux in acetonitrile; (*ii*) condensation with squaric acid in *n*-butanol/pyridine (10% *v*/*v*) under reflux; (*iii*) conversion to the *O*-methoxy-substituted squaraine using methyl trifluoromethanesulfonate in dry dichloromethane under nitrogen atmosphere; (*iv*) and (*v*) nucleophilic substitution with the corresponding amines in dry dichloromethane at room temperature under nitrogen atmosphere, followed by counterion exchange with 14% aqueous potassium iodide; (*vi*) deprotection of the *N*-Boc-ethylenediamine derivative using trifluoroacetic acid, followed by counterion exchange with 14% aqueous potassium iodide.

Aminosquaraine dyes **1**–**8** (Scheme 1) were synthesized using an expeditious approach previously developed by our group [18]. The synthesis began with the preparation of benzothiazolium quaternary ammonium salts bearing *N*-butyl, -hexyl, and -decyl chains. These salts were subsequently reacted with half an equivalent of squaric acid to afford the corresponding zwitterionic squaraines, which were then methylated with methyl trifluoromethanesulfonate to yield the *O*-methyl ether derivatives. These intermediates were further reacted with amines of different nature: dye **1** was obtained using ammonia, dyes **2** and **3** were synthesized using methylamine, dye **4** was prepared with ethanolamine and dye **5** with diethanolamine, whereas compounds **6**–**8** were obtained using *N*-Boc-protected ethylenediamine to prevent disubstitution; following nucleophilic substitution, acidic treatment readily removed the protecting group, affording the corresponding free terminal amines. The obtained aminosquaraines were finally converted to their iodide salts via counterion exchange, a strategy that facilitates precipitation and subsequent purification by recrystallization.

The design of these molecules aimed to modulate their biological performance through structural variation: the pendant *N*-alkyl chains were intended to tune hydro-/lipophilicity, potentially affecting cellular uptake, while amino and hydroxyl groups at the squaric ring may improve aqueous solubility and promote hydrogen-bonding interactions with biological targets, thereby potentially enhancing biological activity [19]. The benzothiazole scaffold is present in clinically approved drugs such as riluzole, and although not widely represented in approved anticancer agents, it has been extensively investigated in oncology owing to its recognized pharmacological significance [20,21].

### 2.2. Biological Evaluation

#### 2.2.1. Metabolic Activity Evaluation

The cytotoxic effects of the aminosquaraines **1**–**8** were evaluated in breast (MCF-7 and T47D) and prostate (LNCaP) cancer cell lines using the MTT assay, as previously described [17]. The choice of these cell lines is based on their clinical relevance, as breast cancer remains the most frequently diagnosed cancer worldwide [22], while prostate cancer is the most commonly diagnosed malignancy in men and the second leading cause of cancer-related mortality [23,24]. Untreated cells and 5-fluorouracil (5-FU) were used as negative and positive controls, respectively. Normal human dermal fibroblasts (NHDF) were included as a non-cancer model to assess tumor selectivity.

In a preliminary screening, cells were exposed to a single concentration (30 µM) of each dye for 72 h to assess metabolic activity. All compounds markedly reduced metabolic activity, except for dye **8** in LNCaP and T47D cells, which produced an approximately 50% reduction. Based on these findings, full concentration-response curves (0.01–50 µM) were obtained, and half-maximal inhibitory concentration (IC_50_) values were calculated by sigmoidal fitting; the results are presented in Table 1.

Analysis of the activity of the aminosquaraine dyes against LNCaP prostatic adenocarcinoma cells revealed that dyes **2** and **3**, both functionalized with a methylamino group, were the most prominent in terms of cytotoxic potency. Dye **2** exhibited an IC_50_ value of 0.17 µM and was approximately three times more cytotoxic toward this cancer cell line than toward normal fibroblasts. Dye **3**, although displaying no tumor selectivity (SI of 0.53), also showed strong activity, reducing cellular metabolic activity by 50% at a concentration of 0.74 µM. By contrast, dye **5** demonstrated a more favorable differential cytotoxicity profile; however, nearly twice the concentration was required to achieve the same cytotoxic effect, indicating a comparatively lower potency.

As anticipated [25], among aminosquaraines bearing the same *N*-alkyl group, modification of the amine substituent also had a clear impact on biological activity: for example, when considering molecules bearing *N*-hexyl chains against LNCaP cells, functionalization with a primary amino or methylamino groups resulted in high potency but no selectivity. In contrast, the introduction of ethanolamino, and diethanolamino substituents led, respectively, to decreased cytotoxicity accompanied by a progressive increase in SI ratios. The analogue bearing an ethylenediamine moiety was not only weakly potent but also failed to exhibit selectivity.

Concerning MCF-7 breast adenocarcinoma cells, a cytotoxicity profile relatively similar to that observed for LNCaP cells was observed. Dye **2** again emerged as the most promising compound, exhibiting high potency (IC_50_ = 0.28 µM), although with a slight reduction in differences in cellular sensitivity toward MCF-7 cells compared with NHDF cells. Once again, despite displaying comparable potency, dye **3** showed poor selectivity. Interestingly, dyes **5** and **6** demonstrated noteworthy cell-dependent response. Although dye **6** was the most potent of these two, it remained less cytotoxic than dyes **2** and **3** overall. Nevertheless, both compounds were approximately 6–7 times more cytotoxic to tumor cells than to normal fibroblasts. Thus, while the introduction of a diethanolamino group (dye **5**) resulted in enhanced selectivity (SI = 7.07), this improvement occurred at the expense of potency when compared with its methylamino and ethanolamino analogues (dyes **3** and **4**, respectively).

Interestingly, the activity of dye **5** in T47D cells revealed an enhanced activity profile. The increased sensitivity of these cells to this compound resulted in a considerably lower IC_50_ value (0.24 µM) and, consequently, in an approximately 24-fold difference in activity compared with non-tumoral cells. In contrast, although significantly less selective (SI values ranging from 1.4 to 2.8), dyes **2** and **3** exhibited strong cytotoxic effects in this cell line, with IC_50_ values of 0.37 and 0.14 µM, respectively.

From a preliminary structure-activity relationship perspective, and considering the overall metabolic activity results, functionalization of the squaric ring with a primary amine or a methylamino group proved to be the most advantageous modifications, yielding the most potent compounds. In contrast, conjugation with ethanolamine and diethanolamine groups reduced potency in LNCaP and MCF-7 cells, although IC_50_ values of a similar order of magnitude were observed in the other tumor cell line. Moreover, when comparing these two derivatives (dyes **4** and **5**), the introduction of the tertiary amine in dye **5** generally resulted in decreased potency relative to aminosquaraine dye **4**, with the exception of T47D cells.

Dyes **6**–**8**, functionalized with an ethylenediamine moiety, generally displayed relatively weak cytotoxic activity, showing lower potency than 5-FU, although compound **7** exhibited comparable or higher activity in LNCaP and T47D cells. Interestingly, within this series, the optimal *N*-alkyl chain length appeared to be *N*-hexyl (dye **7**), which exhibited slightly higher cytotoxicity than the other analogues. In contrast, the derivatives bearing *N*-butyl and especially *N*-decyl chains showed markedly reduced biological activity.

Overall, aminosquaraines **2** and **3** emerged as among the most potent candidates, as both dyes induced pronounced cytotoxic effects across the tested cancer cell lines. However, their therapeutic potential must be interpreted considering their limited tumor preferential activity. Nevertheless, the cytotoxic activity of these compounds is noteworthy when compared with the well-established anticancer agent 5-FU. Relative to this reference drug, aminosquaraines **2** and **3** exhibited markedly higher potency. For example, dye **2** was approximately 46-fold more potent than 5-FU in LNCaP cells and about 6-fold more potent in MCF-7 breast adenocarcinoma cells.

Squaraine-based compounds are known to strongly absorb in the visible region, particularly in the red and near-infrared range of the electromagnetic spectrum [15]. Upon irradiation at these wavelengths, they can generate photocytotoxic effects, making them promising candidates for photodynamic therapy [26]. In this context, it would be particularly interesting to investigate the photodynamic activity of dyes **5**–**8** in future studies, as these molecules exhibited relatively high IC_50_ values across all tested cell lines, which could potentially be reduced upon light activation. In contrast, the remaining aminosquaraines already display pronounced cytotoxicity in the absence of light irradiation and therefore appear less suitable for a photodynamic therapeutic approach.

#### 2.2.2. SubG1 and Cell Cycle Analysis

Considering the results obtained in the MTT assays, the potential of aminosquaraines **2** and **3** to induce cell cycle arrest and apoptosis was further investigated by flow cytometry using propidium iodide (PI) staining, according to the method described by Riccardi and Nicoletti [27]. The antimetabolite 5-FU was used as a positive control due to its well-established ability to induce S-phase cell cycle arrest and trigger apoptotic events associated with DNA damage and antiproliferative effects [17]. Following data acquisition, cellular debris were excluded by defining the region of interest in the FSC/FL3 contour plot (R4 gate). Subsequently, single cells were selected to avoid doublet interference, allowing accurate analysis of DNA content distribution. The percentage of cells in the G0/G1, S, and G2/M phases of the cell cycle was then determined.

**Figure 1:**
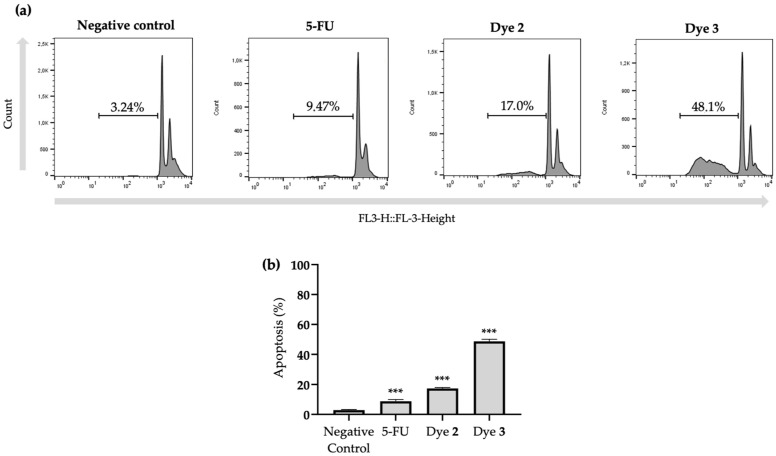
Apoptosis analysis in MCF-7 cells treated with aminosquaraine dyes **2** and **3**. MCF-7 cells were left untreated (negative control) or treated with 5-fluorouracil (5-FU, 10 µM) and aminosquaraines (0.50 µM) for 72 h, followed by propidium iodide (PI) staining and flow cytometry. (**a**) Representative DNA content histograms obtained from PI fluorescence measured in the FL3-H channel. (**b**) Quantitative analysis of subG1 population expressed as mean ± standard deviation from two independent experiments performed in duplicate. Statistical significance was determined using Student’s *t*-test, where *** *p* < 0.001 versus the negative control.

Regarding the quantification of the subG1 population, corresponding to cells presenting fragmented or degraded DNA content, it was observed that 5-FU (10 µM) induced an increase of approximately 6% compared to the negative control (Figure 1).

Remarkably, at a concentration twenty times lower than that of 5-FU, the aminosquaraines **2** and **3** produced considerably more pronounced effects. After 72 h of incubation, the subG1 population of MCF-7 cells reached 17% for dye **2** and, strikingly, approximately 48% for dye **3**. These results indicate the induction of apoptotic-like cell death by these compounds, as the accumulation of cells in the subG1 region is a well-established marker of DNA fragmentation potentially associated with apoptosis. SubG1 analysis alone does not fully reflect the underlying apoptotic pathways; therefore, these findings should be interpreted as preliminary rather than definitive evidence of caspase-regulated apoptosis, a highly regulated and controlled mechanism of cell death, characterized by DNA cleavage, chromatin condensation, and membrane integrity preservation, which generally prevents the release of intracellular pro-inflammatory contents [28,29]. Consequently, the potential ability of these dyes to promote apoptosis rather than uncontrolled necrotic death may represent an advantageous feature from a therapeutic perspective, as it suggests the potential to eliminate cancer cells while minimizing inflammatory responses and collateral damage to surrounding tissues.

**Figure 2:**
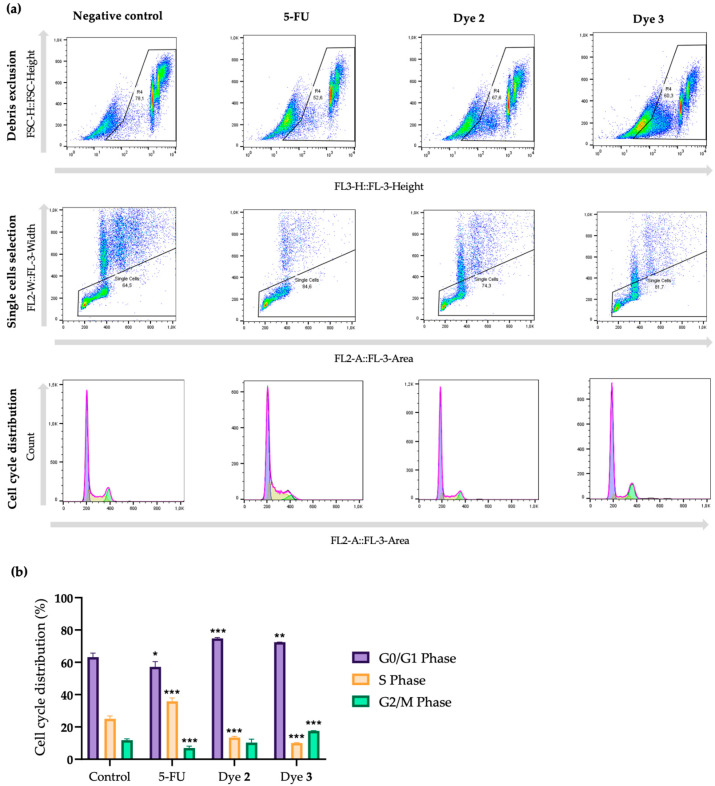
Effects of aminosquaraine dyes **2** and **3** on the cell cycle distribution of MCF-7 cells. Cells were treated with amine-squaraine dyes (0.50 µM) or with the positive control 5-fluorouracil (5-FU, 10 µM) for 72 h, followed by propidium iodide (PI) staining and flow cytometry analysis of DNA content. (**a**) Representative flow cytometry plots illustrating the gating strategy and cell cycle analysis. In the first step, cellular debris was excluded based on FSC/SSC parameters. Then, single cells were selected to eliminate doublets. Finally, DNA content histograms were generated, allowing the determination of the proportion of cells distributed across the G0/G1, S, and G2/M phases of the cell cycle. (**b**) Quantitative analysis of the cell cycle distribution, expressed as mean ± standard deviation of two independent experiments performed in duplicate. Statistical significance was evaluated using Student’s *t*-test (* *p* < 0.05, ** *p* < 0.01, *** *p* < 0.001 versus negative control).

Regarding the cell cycle distribution, as expected, the antimetabolite 5-FU induced S-phase cell cycle arrest, resulting in a reduction in the percentage of cells in the G0/G1 and G2/M phases compared with the negative control (Figure 2). Considering the antiproliferative activity of dyes **2** and **3**, a clear increase in the proportion of cells in G0/G1 was observed, averaging about 20%, suggesting a potential interference with cell cycle progression at the G1 checkpoint. Consequently, dye **2** caused a significant reduction in the number of events in the S phase, which may indicate inhibition of DNA biosynthesis or impaired progression into this phase. In contrast, dye **3** induced a significant increase in the proportion of cells in the G2/M phase, suggesting disruption of cell cycle progression at later stages, potentially associated with DNA damage or a checkpoint-mediated arrest.

#### 2.2.3. Cellular Uptake

**Figure 3:**
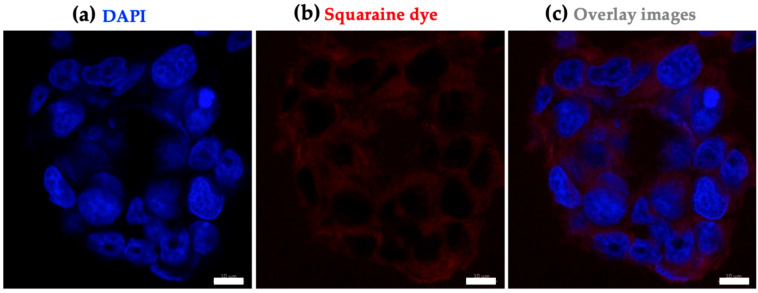
Fluorescence microscopy analysis of the intracellular localization of aminosquaraine dye **3** in MCF-7 cells. Cells were incubated with dye **3** (10 µM) for 6 h, followed by fluorescence imaging. (**a**) Nuclear staining with 2-(4-amidinophenyl)-1*H*-indole-6-carboxamidine (DAPI; excitation 405 nm, emission 499 nm). (**b**) Fluorescence signal of dye **3** (excitation 633 nm, emission 697 nm). (**c**) Overlay of the nuclear stain and squaraine fluorescence channels. Scale bar: 10 µm.

After identifying aminosquaraine dye **3** as the most cytotoxic and effective apoptosis inducer among the selected dyes, its intracellular distribution was investigated by fluorescence microscopy in MCF-7 cells. As shown in Figure 3, the nuclear staining with DAPI clearly delineates the nuclei of MCF-7 cells. In contrast, the fluorescence signal corresponding to dye **3** is predominantly observed in the cytoplasmic region. Importantly, squaraine fluorescence does not significantly overlap with the nuclear DAPI signal, indicating that the dye does not preferentially accumulate within the cell nucleus. The overlay images further confirm that dye **3** is mainly localized in the cytoplasm and perinuclear regions, suggesting that its cellular targets are likely associated with cytoplasmic or organelle structures rather than nuclear components.

This intracellular distribution is consistent with that reported for several squaraine-based molecules [30,31,32], which display strong affinity for intracellular organelles due to their lipophilic and cationic nature. Such localization may contribute to the cytotoxic and pro-apoptotic activity of this compound, as accumulation in mitochondria or other metabolically active organelles may induce cellular stress and trigger apoptotic pathways, consistent with the increased sub-G1 population detected by flow cytometry.

## 3. Materials and Methods

### 3.1. Chemistry

Reagents and solvents were purchased from commercial sources and were of analytical grade. Solvents were dried whenever necessary using standard procedures and freshly distilled prior to use. Reactions were monitored by thin-layer chromatography (TLC) using 0.25 mm Al-backed silica-gel plates (Merck 60 F254, Merck, Rahway, NJ, USA). UV-Vis spectra were performed on a Perkin Elmer Lambda 25 instrument (PerkinElmer, Thane, India); λ_max_ in nm. Molar absorption coefficient was calculated using the Beer-Lambert law. IR spectra were recorded in a Unicam Research Series FT-IR spectrophotometer (Thermo Fisher Scientific, Waltham, MA, USA); ν_max_ in cm^−1^ and intensities are described as strong (s), medium (m) or weak (w). ^1^H and ^13^C NMR spectra were acquired at room temperature on a Brüker Avance III 400 MHz spectrometer (^1^H NMR at 400.13 MHz and ^13^C NMR at 100.62 MHz, Bruker, Billerica, MA, USA). Deuterated dimethyl sulfoxide (DMSO-*d*_6_) was used as solvent and internal standard. Coupling constants (*J* values) are reported in hertz (Hz) and splitting multiplicities are described as singlet (s), broad singlet (br s), doublet (d), triplet (t), broad triplet (br t), q (quartet) or m (multiplet). High resolution electrospray ionization time-of-flight mass spectra (HRESI-TOFMS) were obtained on a Micromass VG AutoSpec M spectrometer (Micromass, Manchester, UK). Molecular ions are indicated as [M]^+^. Aminosquaraine dyes **1** [33], **3** [18], **4** [34], **5** [35], and **6**–**8** [36] were prepared as previously described. To the best of our knowledge, compound **2** is here described for the first time (please see the next section and Supplementary Materials).

2-[3-(3-Butyl-3H-benzothiazol-2-ylidenemethyl)-2-methylamino-4-oxocyclobut-2-enylidenemethyl]-3-butylbenzothiazol-3-ium Iodide (**2**)
Yield: 85%. Purple crystals. UV/Vis (MeOH/CH_2_Cl_2_, 99:1) λ_max_ (log ε): 660 (5.28). IR (KBr) ν_max_: 1630 (s), 1477 (m), 1354 (s), 1311 (s), 1254 (m), 1153 (w), 1107 (w), 1028 (w). ^1^H NMR (400.13 MHz, DMSO-*d*_6_) δ: 8.76 (1H, br s, NH), 7.98 (1H, d, *J* = 8.0, ArH), 7.92 (1H, d, *J* = 8.0, ArH), 7.70 (1H, d, *J* = 8.4, ArH), 7.64 (1H, d, *J* = 8.4, ArH), 7.55–7.47 (2H, m, ArH), 7.39–7.30 (2H, m, ArH), 6.17 (1H, s, CH=C), 6.01 (1H, s, CH=C), 4.36 (2H, br t, *J* = 7.4, NCH_2_(CH_2_)_2_CH_3_), 4.27 (2H, br t, *J* = 7.4, NCH_2_(CH_2_)_2_CH_3_), 3.30 (3H, br d, *J* = 4.8, NHCH_3_), 1.74–1.67 (4H, m, NCH_2_CH_2_CH_2_CH_3_), 1.48–1.36 (4H, m, N(CH_2_)_2_CH_2_CH_3_), 0.94 (6H, t, *J* = 7.4, N(CH_2_)_3_CH_3_). ^13^C NMR (100.62 MHz, DMSO-*d*_6_) δ: 173.8, 164.5, 161.6, 160.0, 157.3, 156.6, 141.2, 128.3, 128.1, 125.4, 125.0, 123.4, 123.1, 114.0, 113.5, 87.2, 87.0, 46.8, 46.0, 30.8, 29.9, 29.5, 19.9, 14.2, 14.1. HRESI-TOFMS *m*/*z*: 502.19846 ([M – I]^+^, C_29_H_32_N_3_OS_2_^+^; calc. 502.19813).

### 3.2. Biological Evaluation

Human breast cancer cell lines (MCF-7 and T47D), prostate cancer cells (LNCaP), and normal human dermal fibroblasts (NHDF) were obtained from the American Type Culture Collection (ATCC, Manassas, VA, USA). Unless otherwise stated, all analytical grade chemicals, assay reagents, culture media, and supplements were purchased from Sigma-Aldrich (St. Louis, MO, USA). All experiments were conducted under dark conditions.

#### 3.2.1. Cell Culture

Cells were grown in 75 cm^2^ culture flasks under standard culture conditions (37 °C, humidified atmosphere with 5% carbon dioxide). NHDF cells were maintained in Roswell Park Memorial Institute (RPMI) medium supplemented with 10% fetal bovine serum (FBS), 2.0 mM L-glutamine, 10 mM 2-[4-(2-hydroxyethyl)piperazin-1-yl]ethanesulfonic acid (HEPES), 1.0 mM sodium pyruvate, and 1% antibiotic-antimycotic mixture (10,000 units/mL penicillin G, 10 mg/mL streptomycin, and 25 µg/mL amphotericin B). LNCaP and T47D cells were cultured in RPMI medium containing with 10% FBS and 1% antibiotic solution (10,000 units/mL penicillin G and 10 mg/mL streptomycin). MCF-7 cells were maintained in high-glucose Dulbecco’s Modified Eagle’s Medium (DMEM) supplemented with 10% FBS and 1% antibiotic-antimycotic solution.

The culture medium was replaced every 2–3 days until cells reached approximately 90–95% confluence. Cells were then harvested by mild trypsinization, and viable cells were determined using the trypan blue exclusion assay prior to dilution in the appropriate complete culture medium for succeeding experiments.

#### 3.2.2. Preparation of Compounds Solution

The studied compounds were dissolved in dimethyl sulfoxide to prepare 10 mM stock solutions and stored at 4 °C. Working solutions were prepared from these stocks at the desired concentrations (30 µM for preliminary studies and 0.01, 0.1, 1, 10, 25, and 50 µM for concentration–response studies) by dilution in complete culture medium immediately before each experiment. The final dimethyl sulfoxide concentration did not exceed 1%, which had no significant effect on cell viability.

#### 3.2.3. MTT Assay

Metabolic activity was assessed by quantifying the reduction of 3-(4,5-dimethylthiazol-2-yl)-2,5-diphenyltetrazolium bromide (MTT), according to a previously described procedure [17]. Briefly, cells were seeded in 96-well plates (Nunc, Apogent, Roskilde, Denmark) at a density of 2 × 10^4^ cells/mL in complete culture medium and allowed to adhere for 48 h. The cells were then exposed to the test compounds at the specified concentrations for 72 h. Untreated cells were used as the negative control, while 5-FU was used as a positive control. At the treatment period, the culture medium was carefully aspirated, and the cells were rinsed with phosphate-buffered saline (PBS; NaCl 137 mM, KCl 2.7 mM, Na_2_HPO_4_ 10 mM, KH_2_PO_4_ 1.8 mM; pH 7.4). Subsequently, 100 µL of MTT solution (5 mg/mL in serum-free medium) was added to each well, followed by incubation at 37 °C for 4 h to allow the formation of formazan crystals. The supernatant was then removed, and the insoluble formazan product was dissolved in DMSO. Absorbance was recorded at 570 nm using an xMark™ microplate reader (Bio-Rad, Hercules, CA, USA) using Microplate Manager 6.3 software. Cell viability was calculated as a percentage relative to untreated control cells.

#### 3.2.4. Flow Cytometry

Apoptosis induction and cell cycle distribution were evaluated by PI staining followed by flow cytometry. MCF-7 cells were trypsinized at near confluence, counted, and seeded in 6-well plates (2 mL; 6 × 10^4^ cells/mL). After 48 h to allow cell adhesion, the culture medium was replaced with fresh medium containing the test solutions (3 mL per well; 5 mL for the negative control). Untreated cells served as the negative control, while 5-FU was used as the positive control.

After 72 h of incubation, the culture supernatant was collected into ice-cooled centrifuge tubes, together with the PBS washing solution, the trypsinized cells, and an additional PBS wash to ensure complete cell recovery. The resulting cell suspension was centrifuged, and the pellet was resuspended in 0.5 mL PBS and fixed with 4.5 mL of ice-cold 70% ethanol. Cells were stored at −20 °C until flow cytometry analysis.

Prior to analysis, fixed cells were washed with PBS and centrifuged to remove the ethanolic supernatant. The pellet was resuspended in 0.5 mL PBS and 0.5 mL DNA extraction buffer (192 mL of 0.2 M sodium phosphate and 8 mL of 0.1% Triton X-100, pH 7.8). After 5 min of incubation, cells were centrifuged again, the supernatant discarded, and the pellet resuspended in 0.3 mL staining solution containing PI (20 µg/mL) and DNase-free RNase (0.2 mg/mL) in PBS. Samples were incubated for at least 30 min in the dark.

A minimum of 20,000 events per sample were acquired using a FACSCalibur flow cytometer (488 nm laser line, BD Biosciences, Milpitas, CA, USA). Data acquisition and apoptosis analysis were performed using BD CellQuest™ Pro 4.0.2 software. Debris and necrotic cells were excluded by gating out events with lower forward scatter (FSC) and reduced PI fluorescence (FL3) relative to hypodiploid apoptotic cells. Two independent experiments were performed.

#### 3.2.5. Confocal Fluorescence

The subcellular localization of aminosquaraine dye **3** was assessed by confocal fluorescence microscopy (LSM 710, Zeiss, Oberkochen, Germany). MCF-7 cells were seeded at a density of 5 × 10^4^ cells/mL in 24-well plates containing 10 mm coverslips pre-treated with poly-L-lysine (Sigma-Aldrich). After 48 h of incubation, cells were treated with dye **3** (1.0 and 10 µM) for 6 h. Following incubation, cells were washed twice with PBS and fixed with 4% paraformaldehyde for 10 min. Nuclei were stained with DAPI (1 µM) for 10 min, followed by three washes with PBS. Coverslips were mounted and samples were analyzed using a confocal laser scanning microscope (LSM 710 AxioObserver; Carl Zeiss, Jena, Germany) equipped with a 63× oil immersion objective (Plan-Apochromat, NA 1.4, oil DIC M27, Zeiss, Oberkochen, Germany). For visualization, dye **3** was excited at 633 nm with emission collected at 697 nm, while DAPI was excited at 405 nm with emission detected at 499 nm. Image processing was performed using ZEN 2.1 SP3 FP3—version 14.0.20.201 software.

#### 3.2.6. Statistics

MTT and flow cytometry assays were performed in quintuplicate and triplicate, respectively. Data acquired in flow cytometry studies are expressed as mean values ± standard deviation and correspond to at least two independent assays. Statistical significance between groups was defined as *p* < 0.05. IC_50_ values were derived from dose-response curves by sigmoidal regression analysis with a 95% confidence level. Selectivity indices were determined as the ratio between IC_50_ values obtained in the NHDF cell line and those obtained in the corresponding tumor cell lines. For flow cytometry data, differences in the cell viability results were assessed for statistical significance at the 95% confidence level.

## 4. Conclusions

The squaraine dye scaffold is highly versatile, allowing extensive structural modification of these molecules. Based on our previous experience, the introduction of amine groups into this core significantly enhances their intrinsic cytotoxicity compared to non-substituted analogues. In the present study, a series of eight benzothiazole-derived aminosquaraines was investigated, with particular emphasis on the impact of *N*-alkyl chain length and the nature of the amine substituent on their in vitro biological activity.

From a structural perspective, several trends can be highlighted: the incorporation of a methylamino group into the central core led to a notable increase in cytotoxicity; in contrast, elongation of the *N*-alkyl chain to decyl resulted in a reduction in biological activity, with the *N*-decyl-substituted dye being the least cytotoxic squaraine in the series. Functionalization with ethanolamino and ethylenediamino groups yielded compounds with significant activity, although not as pronounced as that observed for the methylamino derivatives.

Among the most promising dyes, particularly **3**, apoptosis was identified as the predominant mechanism of cell death in the studied cell model, accompanied by marked antiproliferative effects through the induction of cell cycle arrest. Fluorescence imaging studies further demonstrated that this last aminosquaraine is primarily localized in the cytoplasm, potentially associated with specific organelles, with reduced nuclear accumulation.

Overall, while the most cytotoxic derivatives highlight the potential of these compounds as chemotherapeutic agents, the less cytotoxic analogues may represent promising candidates for photodynamic therapy applications, where lower dark toxicity combined with photoactivation is desirable.

## Figures and Tables

**Table 1:** Half-maximal inhibitory concentration values (IC_50_, µM) of 5-FU and aminosquaraines **1**–**8** against NHDF, MCF-7, T47D and LNCaP cells, and their corresponding selectivity index (SI) relative to non-cancer NHDF cells. Data are representative of at least two independent experiments.

	Cell Lines						
Compound	NHDF	LNCaP		MCF-7		T47D	
	IC_50_	IC_50_	SI	IC_50_	SI	IC_50_	SI
5-FU	3.41	7.80	0.44	1.71	1.99	1.04	3.28
**1**	0.57	1.16	0.49	1.20	0.48	0.24	2.38
**2**	0.52	0.17	3.06	0.28	1.86	0.37	1.41
**3**	0.39	0.74	0.53	0.42	0.93	0.14	2.79
**4**	2.27	1.15	1.04	0.63	1.90	0.44	5.16
**5**	5.87	1.41	4.16	0.83	7.07	0.24	24.46
**6**	15.78	10.53	1.50	2.52	6.26	4.46	3.54
**7**	3.38	3.82	0.88	3.18	1.06	0.45	7.51
**8**	13.28	ND ^1^	-	15.85	0.84	28.31	0.47

^1^ ND is not determined.

## Data Availability

The original contributions presented in this study are included in the article. Further inquiries can be directed to the corresponding authors.

## Supplementary Materials

The following supporting information can be downloaded at: https://www.mdpi.com/article/10.3390/molecules31091537/s1, Figure S1: ^1^H NMR spectrum of **2** in DMSO-*d*_6_; Figure S2: ^13^C NMR spectrum of **2** in DMSO-*d*_6_.
