# Supplementary material for: In Vitro Antiproliferative Effects of Benzothiazole-Based Aminosquaraine Dyes Against Cancer Cell Lines
Source: Molecules. 2026 May 6;31(9):1537. doi: 10.3390/molecules31091537 (PMC13165350; doi:10.3390/molecules31091537)
Supplement: Supplementary file 1 [file molecules-31-01537-s001.zip › molecules-4265106-supplementary.pdf]

In Vitro Antiproliferative Effects of Benzothiazole-Based Aminosquaraine Dyes Against Cancer Cell Lines

Elisabete Alves [1], João L. Serrano [2], Ahmed Al-Najada [3], Sara Cegonho [1], Vânia Graça [4], Eurico Lima [4], Alexandra Varges [2], Adriana O. Santos [1], Paulo Almeida [2], Paulo F. Santos [4,*] and Samuel M. Silvestre [2,*]

[1] RISE-Health, Faculty of Health Sciences, University of Beira Interior, Avenida Infante D. Henrique, 6201-506 Covilhã, Portugal
[2] RISE-Health, Department of Chemistry, Faculty of Sciences, University of Beira Interior, Rua Marquês de Ávila e Bolama, 6201-001 Covilhã, Portugal
[3] King Abdulaziz City for Science and Technology, P.O. Box 6086, Riyadh 11442, Saudi Arabia
[4] CQ-VR—Chemistry Centre of Vila Real, University of Trás-os-Montes and Alto Douro, Quinta de Prados, 5001-801 Vila Real, Portugal
* Correspondence: psantos@utad.pt (P.F.S.); sms@ubi.pt (S.M.S.)

Supplementary Materials

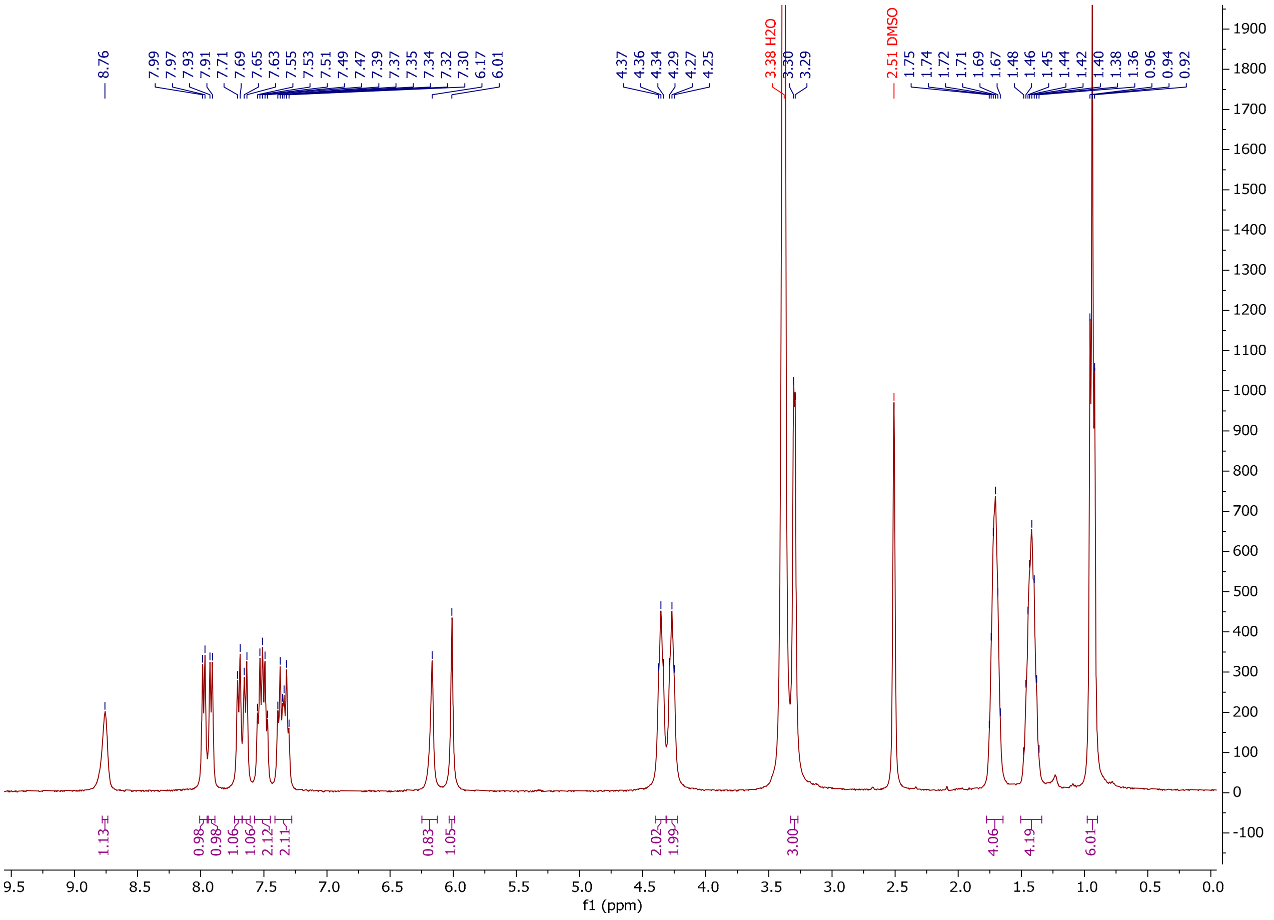

Figure S1. ^{1}**H NMR** spectrum of **2** in DMSO-d_6

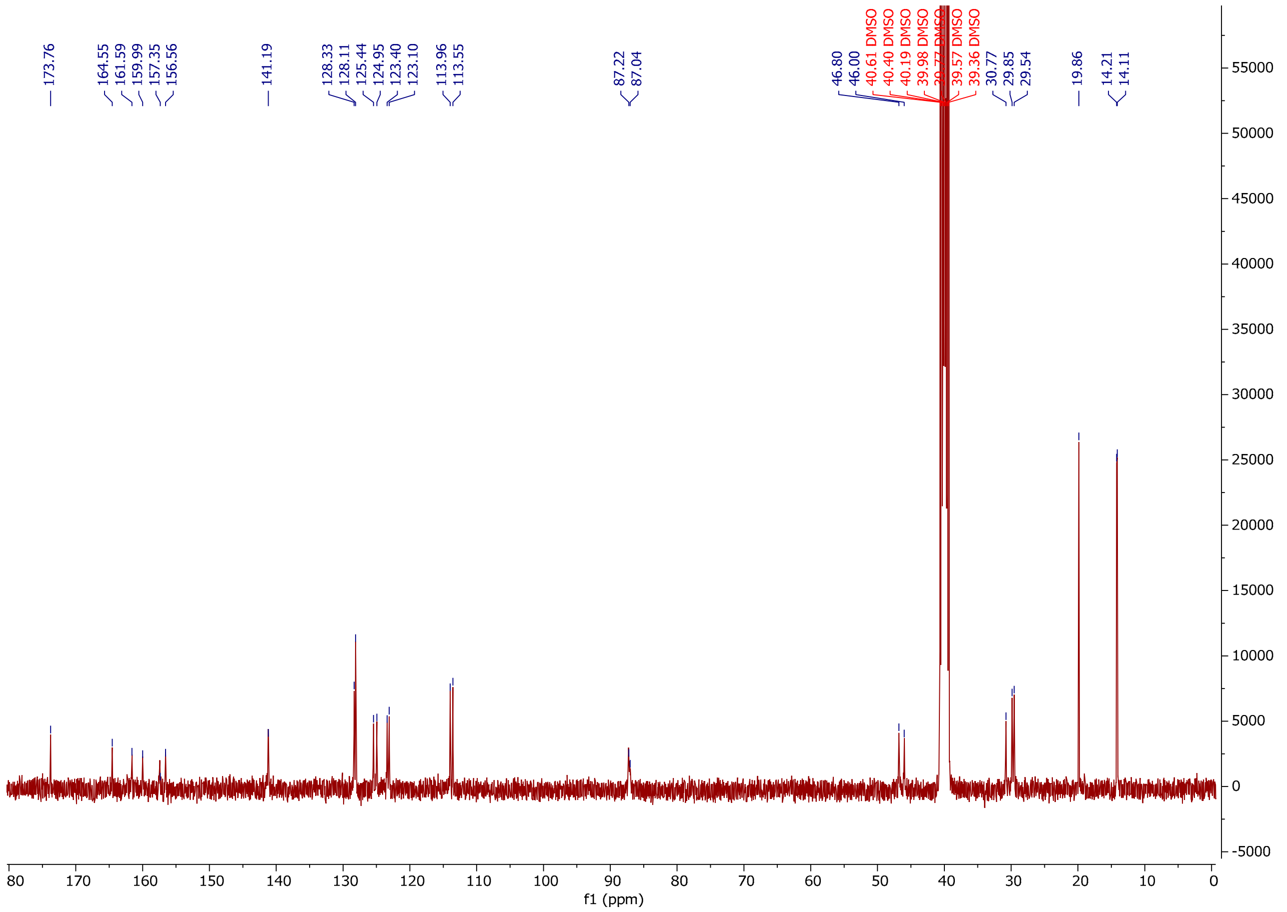

Figure S2. **^{13}C NMR** spectrum of **2** in DMSO-d_6
